# Supplementary material for: Mining mutation contexts across the cancer genome to map tumor site of origin
Source: Nat Commun. 2021 May 24;12:3051. doi: 10.1038/s41467-021-23094-z (PMC8144407; doi:10.1038/s41467-021-23094-z)
Supplement: Supplementary file 1 — Supplementary Information [file 41467_2021_23094_MOESM1_ESM.pdf]

Supplementary Information for "Mining Mutation Contexts across the Cancer Genome to Map Tumor Site of Origin" by Chakraborty et al.

Supplementary Notes

Supplementary Note 1: Some Notes on Model Training Computations

All front-end computations in our study were performed in statistical software R v4.0.2 using the custom package `hidgenclassifier`[1] (https://github.com/c7rishi/hidgenclassifier) implementing the proposed methodologies. Some computational strategies undertaken in training the classifiers described in the main text, through their interfaces in package `hidgenclassifier`, are described below. Note on the outset that any cross-validation performed for tuning model (hyper-) parameters were conducted independently in each training set of the original (outer) 5-fold cross-validation performed for assessing predictive accuracies of the model; no information from tumors outside the training group was used in training. First, the group-lasso regularized multi-logit models were fit using the function `fit_mlogit`, a wrapper for the function cv.glmnet from R package `glmnet`[2], with the penalty parameter λ tuned via independent 20 fold cross-validations with stratified random partitions. Second, the random forest models were trained using the function `fit_rfc`, a wrapper for the function `ranger` from R package `ranger`[3]. The random forest parameter `num.trees` was fixed at 1000 and the parameters mtry and max.depth were tuned by varying `mtry` between $(n_{\text{pred}})^{0.3}$ and $(n_{\text{pred}})^{0.7}$ where n_{pred} denotes the total number of predictors in the model, and max.depth within $\{0, 0.0001, 0.001, 0.01, 0.1, 1, 10\}$; the combination of mtry and max.depth producing the lowest out-of-bag prediction error on the training dataset was retained. Third, the SVM models were trained using the function `fit_svmc` which is a wrapper to the function `mcSVM` from R package `liquidSVM`[4]. Probabilistic multiclass classification with the default all-vs-all comparison strategy and squared error loss was used to fit each SVM. Tuning of the SVM parameters were performed via cross-validation with stratified random partitions, as implemented in `liquidSVM`, with the parameters `max_gamma`, `min_gamma` and `min_lambda` being set to 10^6, 10^{-8} and 10^{-8} respectively. Prior to training each SVM, columns of the corresponding predictor matrix were scaled by twice the column standard deviations. Finally, the deep neural network classifiers were trained through the interface `fit_nnc` which utilizes several functions from R package `keras`[5], tensorflow[6], `caret`[7] and `mlr`[8,9] among others. The deep learning framework was implemented in R using the `keras`[5] and `tensorflow`[6] packages. The neural networks were trained using the Adam optimizer[10] and categorical cross-entropy loss. Following the approach of Jiao et al.[11], we used Bayesian optimization via the `mlrMBO` package[9] to select the following hyperparameters: number of layers (range of values: 0 to 5), number of nodes per layer (range of values: 5 to 1024), learning rate (range of values: [1e-4, 1e-2]), weight decay parameter for L2 regularization (range of values: [1e-3, 0.5]), dropout rate (range of values: [1e-6, 0.5]), and activation function (relu or softmax). The number of epochs was fixed at 50 and batch size was fixed at 128.

Supplementary Figures

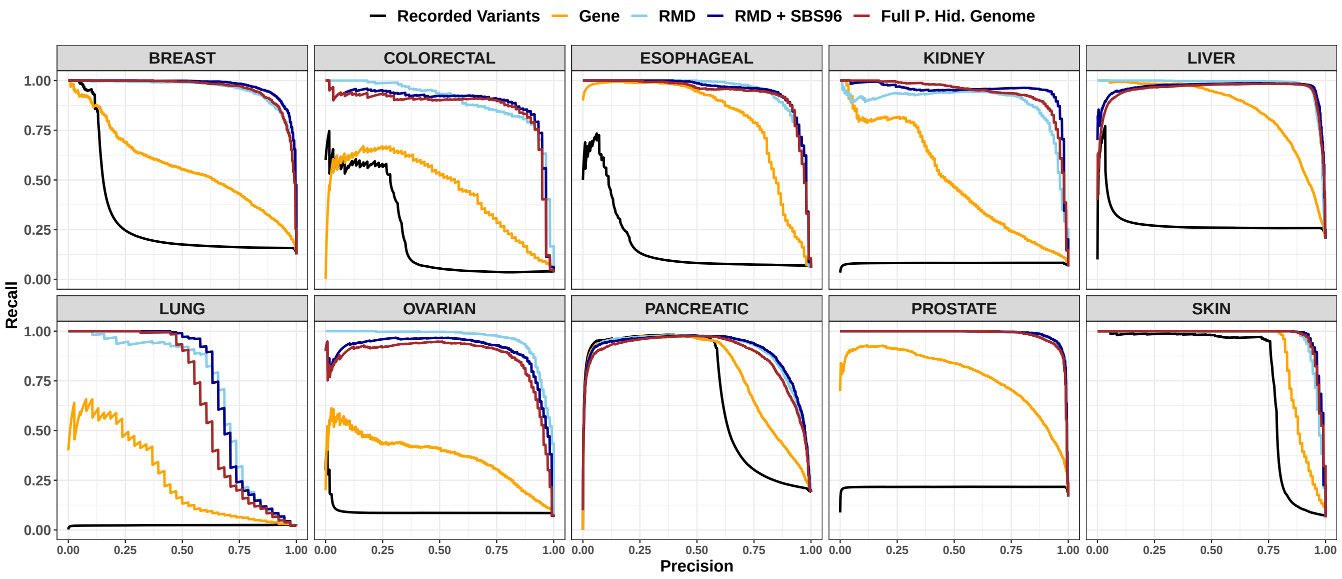

Supplementary Figure 1: Cancer site specific one-vs-rest precision-recall curves comparing cross-validation predictive performances of multinomial logistic classifiers with (i) the baseline recorded variants (Baseline; black lines), (ii) cancer gene indicator (Gene; orange lines), (iii) regional mutation density (RMD; sky blue line), (iv) RMD and nucleotide change signature (RMD + SBS-96; dark blue lines), and (v) all predictors in the full projected hidden classifier (Full P. Hid. Genome; brown lines), all applied to the PCAWG whole genome data.

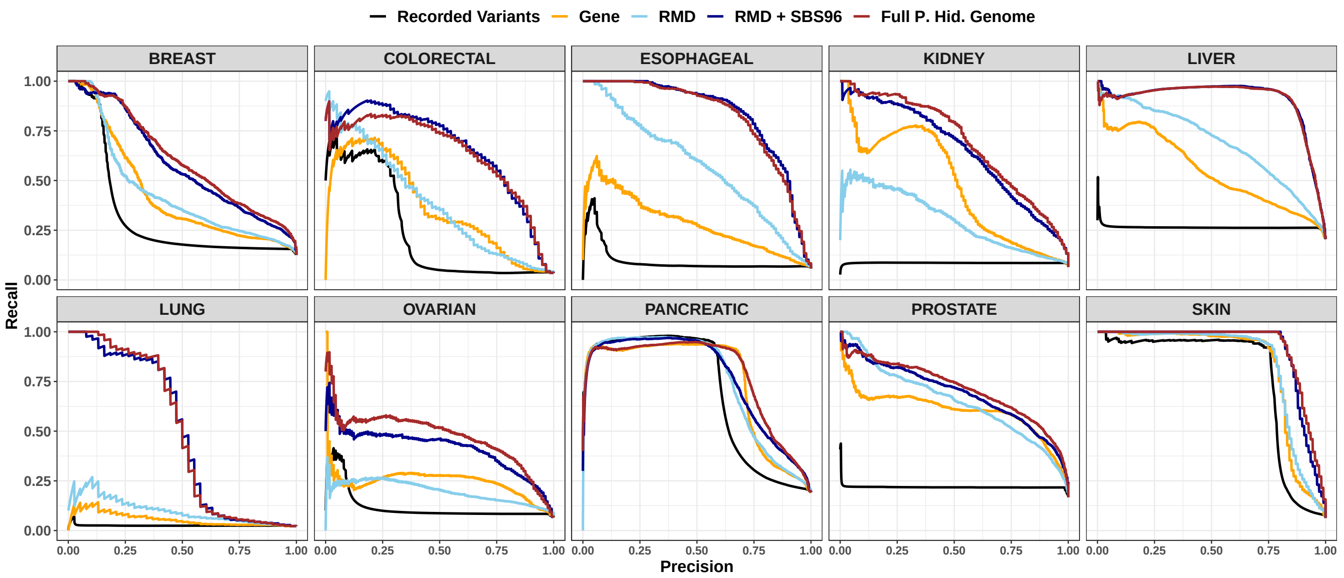

Supplementary Figure 2: Cancer site specific one-vs-rest precision-recall curves comparing cross-validation predictive performances of multinomial logistic classifiers with (i) the baseline recorded variants (Baseline; black lines), (ii) cancer gene indicator (Gene; orange lines), (iii) regional mutation density (RMD; sky blue line), (iv) RMD and nucleotide change signature (RMD + SBS-96; dark blue lines), and (v) all predictors in the full projected hidden classifier (Full P. Hid. Genome; brown lines), all applied to the PCAWG simulated whole exome data.

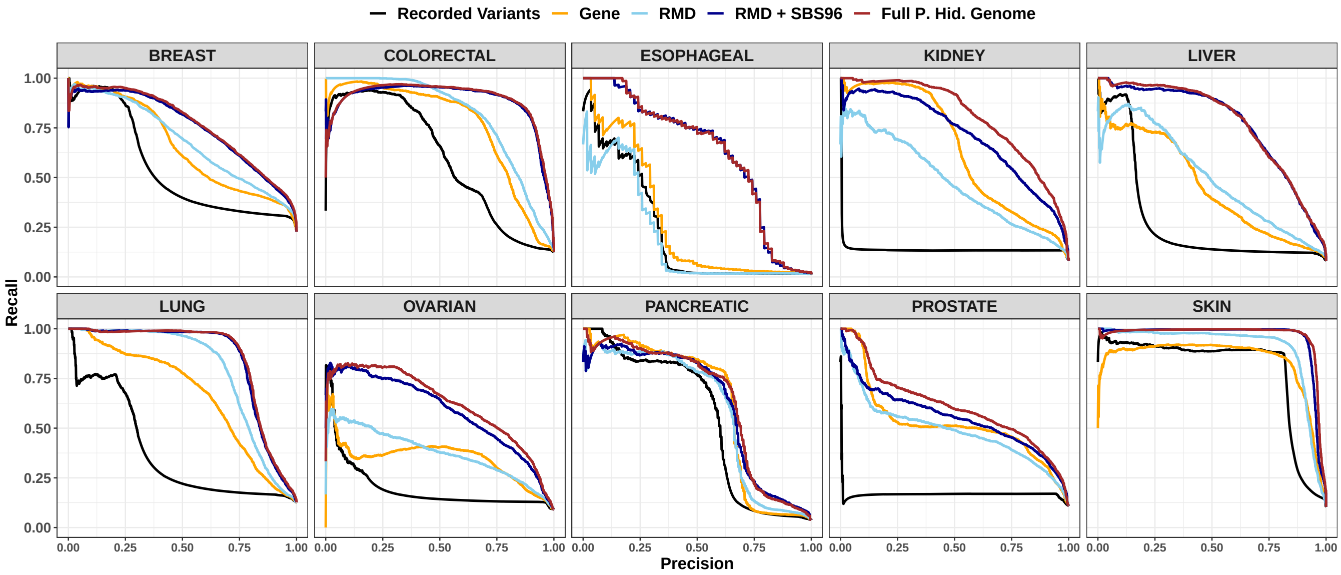

Supplementary Figure 3: Cancer site specific one-vs-rest precision-recall curves comparing cross-validation predictive performances of multinomial logistic classifiers with (i) the baseline recorded variants (Baseline; black lines), (ii) cancer gene indicator (Gene; orange lines), (iii) regional mutation density (RMD; sky blue line), (iv) RMD and nucleotide change signature (RMD + SBS-96; dark blue lines), and (v) all predictors in the full projected hidden classifier (Full P. Hid. Genome; brown lines), all applied to the TCGA whole exome data.

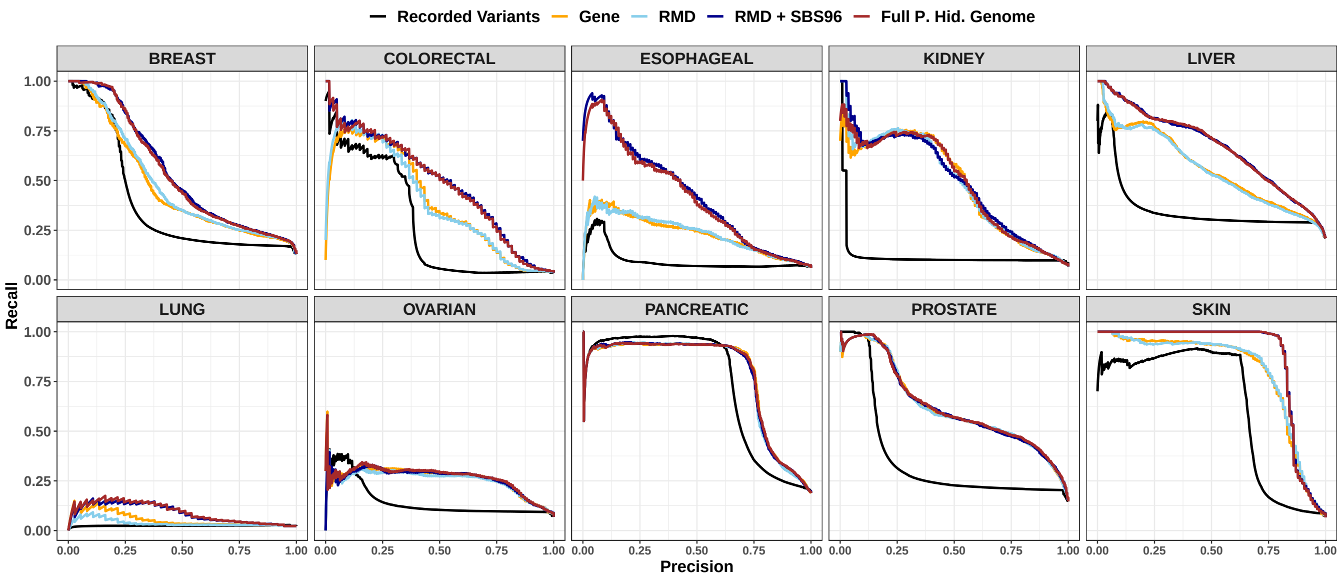

Supplementary Figure 4: Cancer site specific one-vs-rest precision-recall curves comparing cross-validation predictive performances of multinomial logistic classifiers with (i) the baseline recorded variants (Baseline; black lines), (ii) cancer gene indicator (Gene; orange lines), (iii) regional mutation density (RMD; sky blue line), (iv) RMD and nucleotide change signature (RMD + SBS-96; dark blue lines), and (v) all predictors in the full projected hidden classifier (Full P. Hid. Genome; brown lines), all applied to the PCAWG simulated targeted cancer gene panel data.

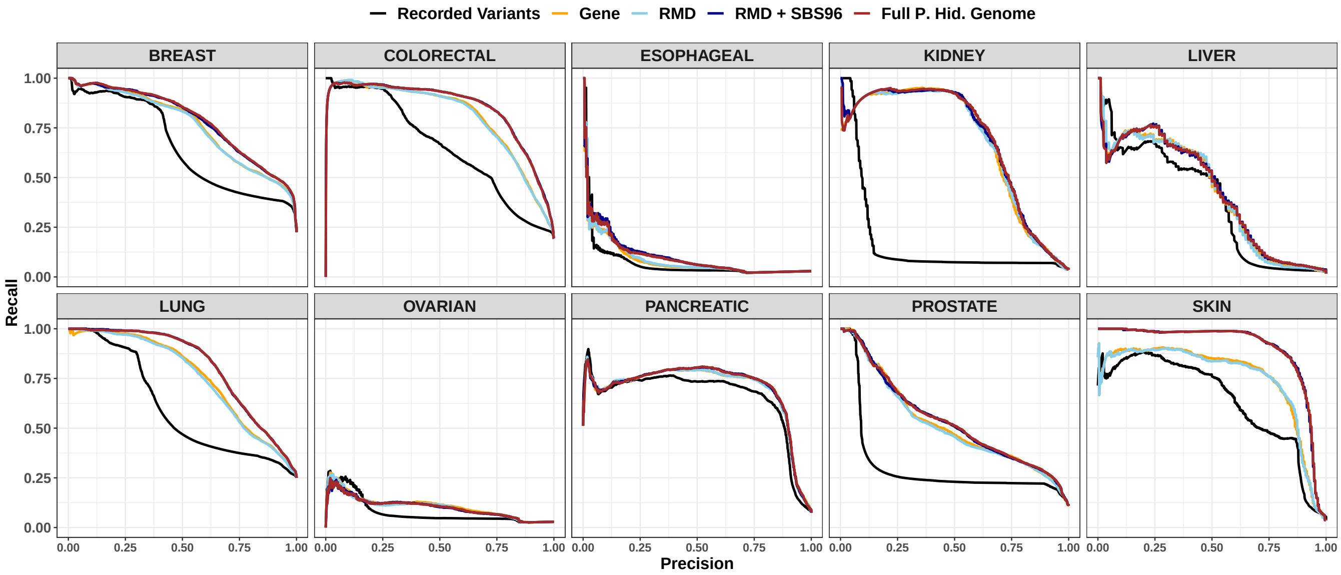

Supplementary Figure 5: Cancer site specific one-vs-rest precision-recall curves comparing cross-validation predictive performances of multinomial logistic classifiers with (i) the baseline recorded variants (Baseline; black lines), (ii) cancer gene indicator (Gene; orange lines), (iii) regional mutation density (RMD; sky blue line), (iv) RMD and nucleotide change signature (RMD + SBS-96; dark blue lines), and (v) all predictors in the full projected hidden classifier (Full P. Hid. Genome; brown lines), all applied to the MSK-IMPACT targeted cancer gene panel data.

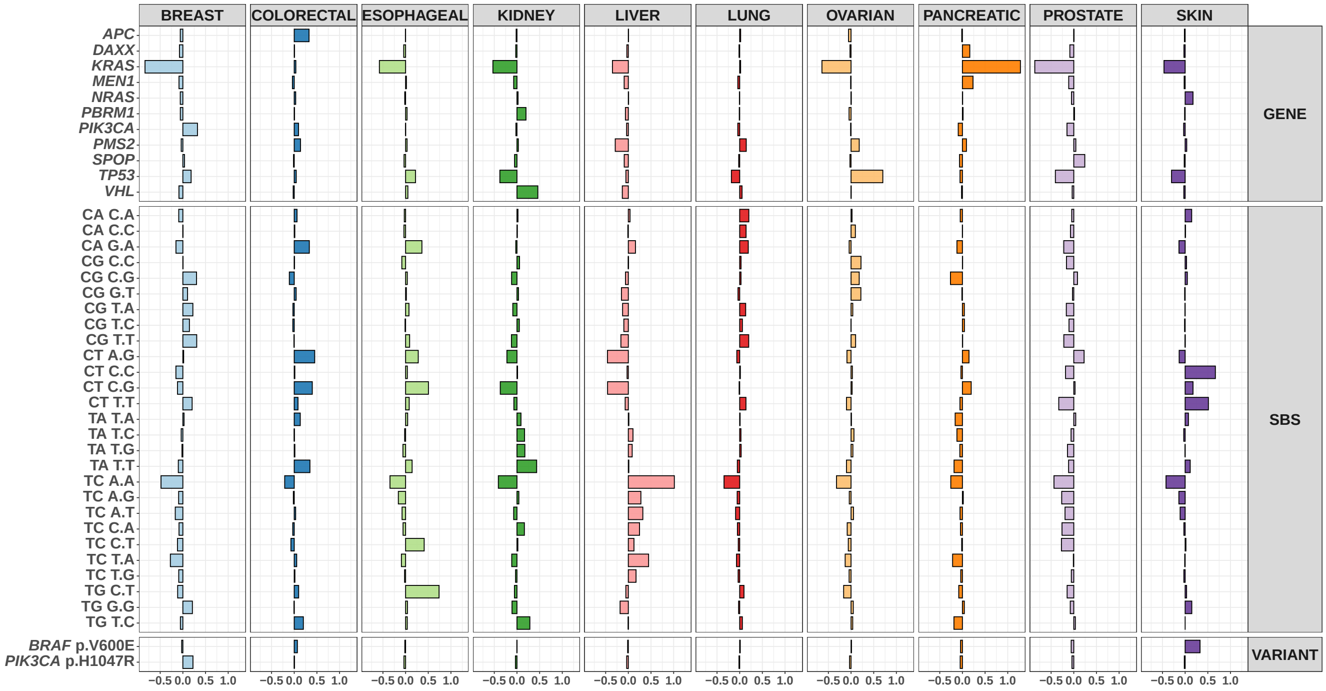

Supplementary Figure 6: Log one-vs-rest odds ratios of top 40 predictors (with largest absolute log odds ratios) in the projected hidden genome classifier applied to complete PCAWG simulated whole-exome dataset. Each bar represents the change in the log odds of a tumor being classified into the corresponding cancer site, relative to not being classified into that site, for a one standard deviation increase in the associated predictor from its mean, while keeping all other predictors fixed at their respective means. Predictors of similar types (variants, genes, SBSs, and chromosome regional indices) are grouped together.

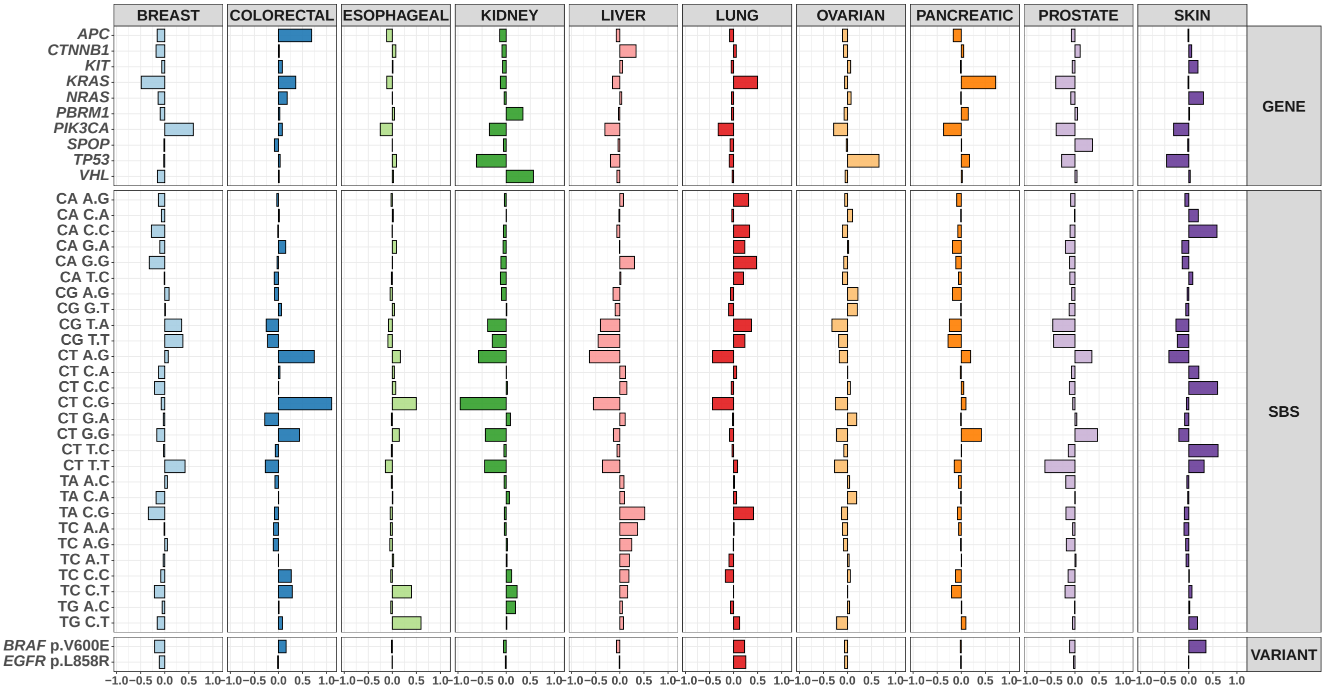

Supplementary Figure 7: Log one-vs-rest odds ratios of top 40 predictors (with largest absolute log odds ratios) in the projected hidden genome classifier applied to complete TCGA whole-exome dataset. Each bar represents the change in the log odds of a tumor being classified into the corresponding cancer site, relative to not being classified into that site, for a one standard deviation increase in the associated predictor from its mean, while keeping all other predictors fixed at their respective means. Predictors of similar types (variants, genes, SBSs, and chromosome regional indices) are grouped together.

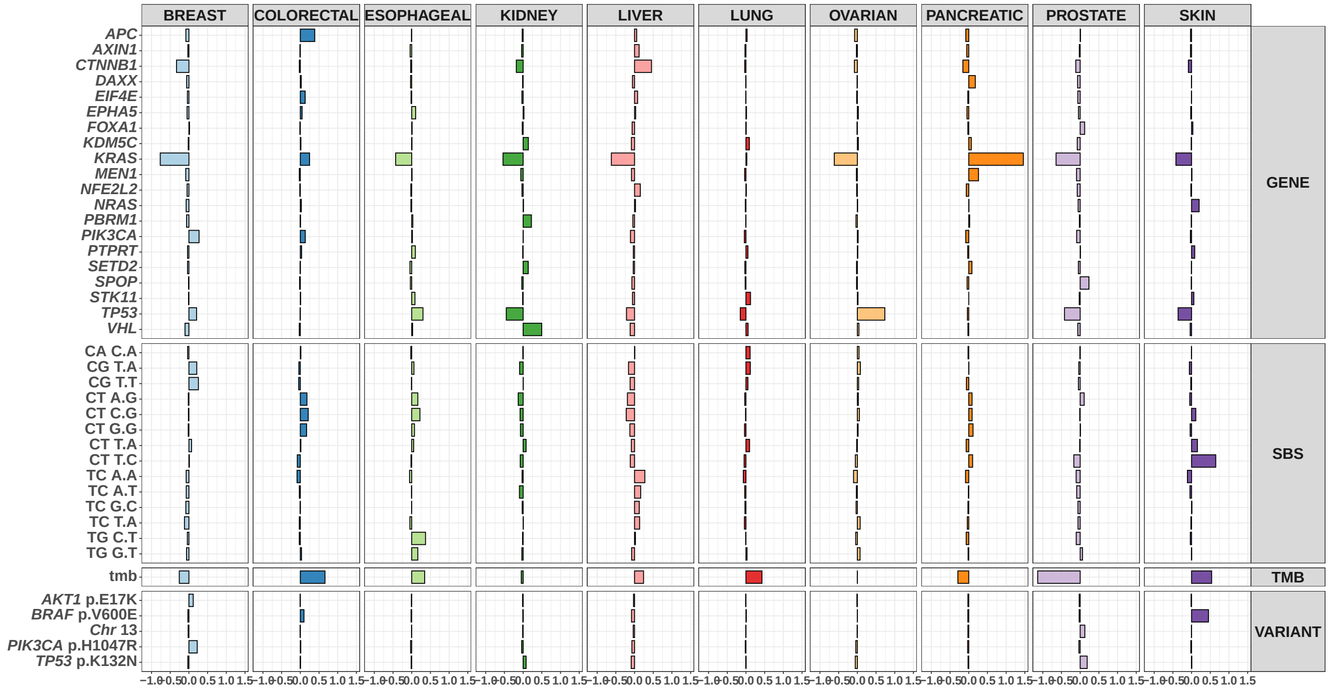

Supplementary Figure 8: Log one-vs-rest odds ratios of top 40 predictors (with largest absolute log odds ratios) in the projected hidden genome classifier applied to complete PCAWG simulated targeted cancer gene panel dataset. Each bar represents the change in the log odds of a tumor being classified into the corresponding cancer site, relative to not being classified into that site, for a one standard deviation increase in the associated predictor from its mean, while keeping all other predictors fixed at their respective means. Predictors of similar types (variants, genes, TMB and SBSs) are grouped together.

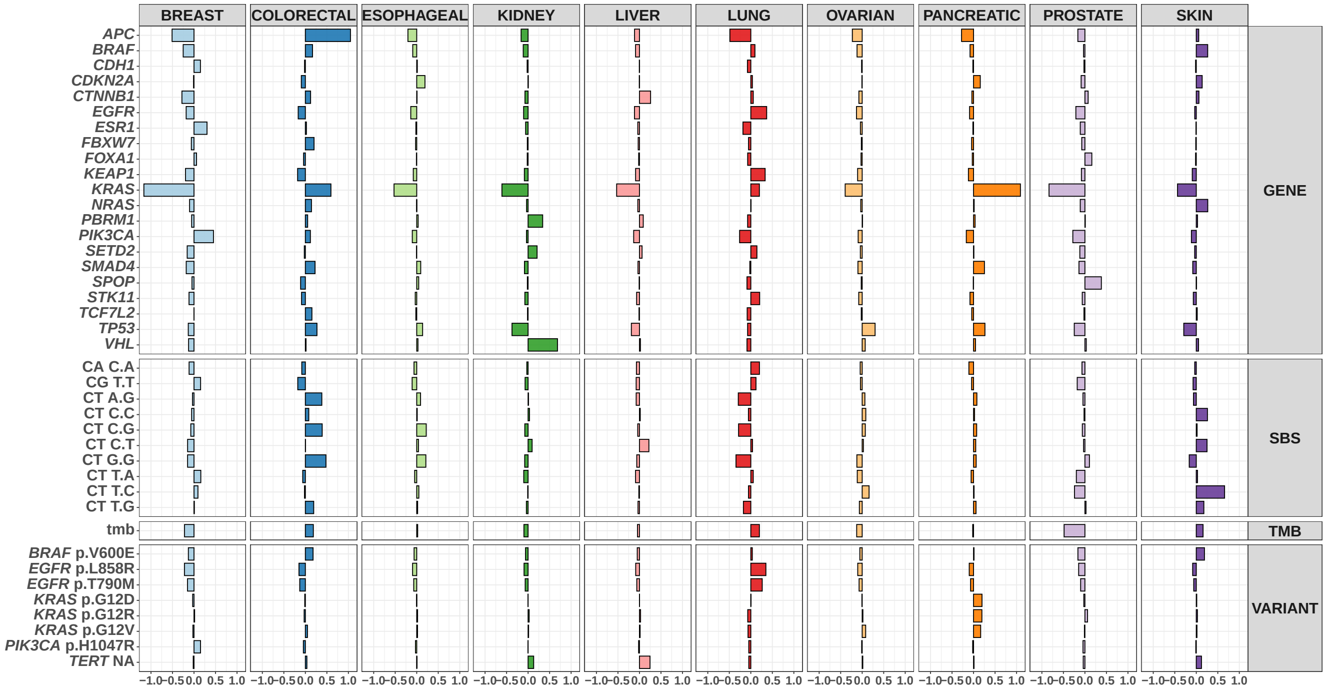

Supplementary Figure 9: Log one-vs-rest odds ratios of top 40 predictors (with largest absolute log odds ratios) in the projected hidden genome classifier applied to complete MSK-IMPACT targeted cancer gene panel dataset. Each bar represents the change in the log odds of a tumor being classified into the corresponding cancer site, relative to not being classified into that site, for a one standard deviation increase in the associated predictor from its mean, while keeping all other predictors fixed at their respective means. Predictors of similar types (variants, genes, SBSs, and chromosome regional indices) are grouped together.

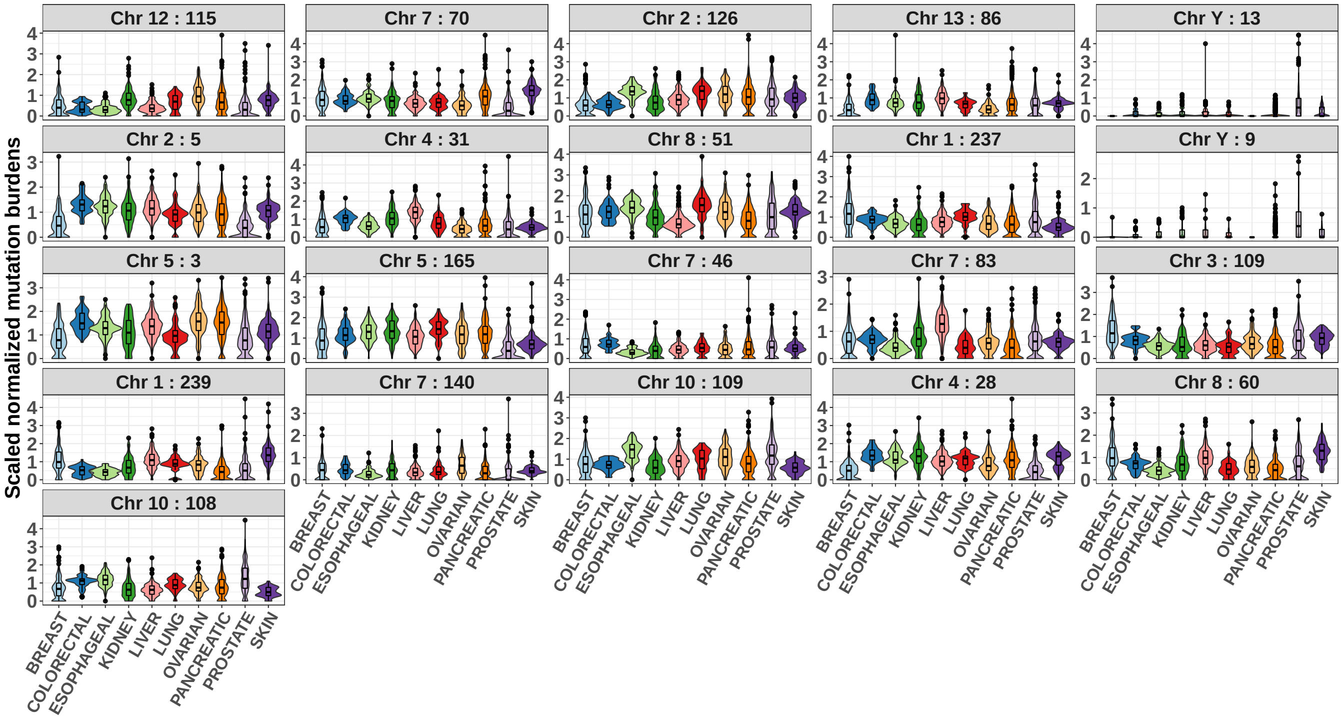

Supplementary Figure 10: Visualizing tissue specificity of regional mutation densities. The per-tumor scaled regional mutation densities (normalized mutation burdens) for the 20 windows displayed in Figure 6 are summarized as box and violin plots separately for each cancer type. For each chromosome region, the differences in relative lengths and positions of the corresponding boxes and violins across different cancer sites demonstrate its tissue specificity. Each boxplot displays the minima and the maxima (outer whiskers) together with the median (the middle-whisker inside the box) and the 25th and 75th percentiles (boundaries of the box) of the corresponding scaled mutational densities across tumors; these site specific statistics are computed from $n = 214$ (BREAST), $n = 60$ (COLORECTAL), $n = 98$ (ESOPHAGEAL), $n = 111$ (KIDNEY), $n = 349$ (LIVER), $n = 38$ (LUNG), $n = 113$ (OVARIAN), $n = 326$ (PANCREATIC), $n = 286$ (PROSTATE), and $n = 107$ (SKIN) tumors separately for each window.

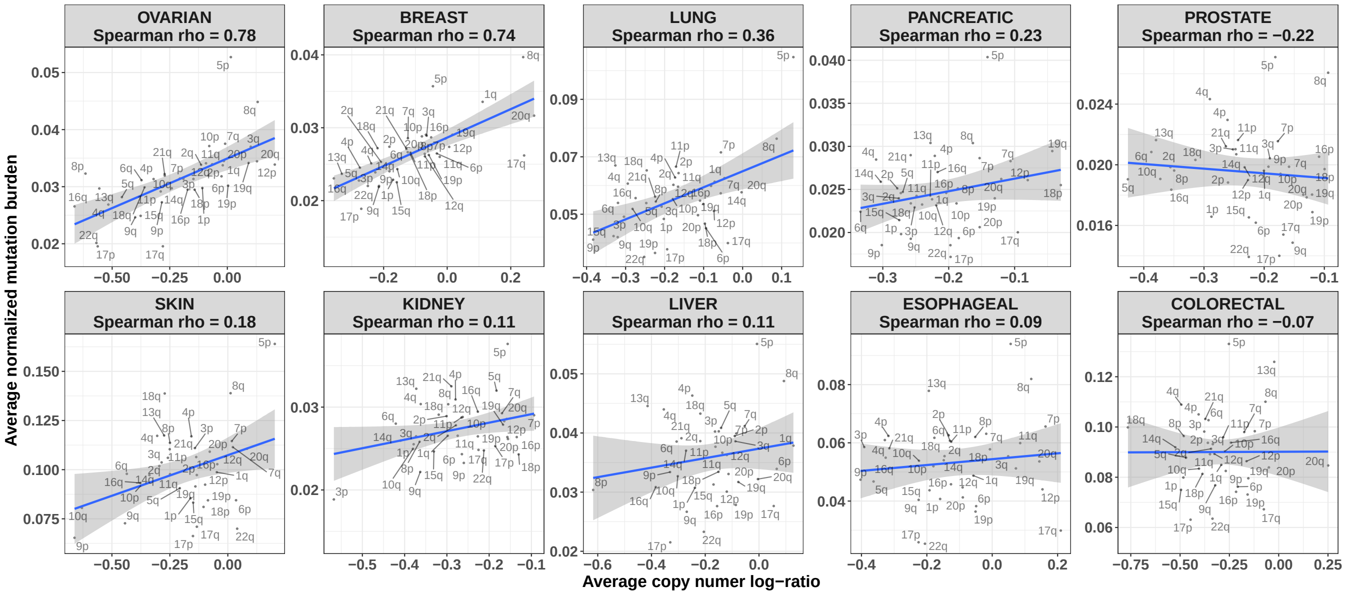

Supplementary Figure 11: Visualizing association between mutation burden and copy number alterations. The average normalized whole genome mutation burden in each chromosome arm is plotted (along the vertical axis) against the corresponding average copy number alteration log ratio (along the horizontal axis) for different cancer sites. The error band in each plot corresponds to 95% prediction intervals for average normalized mutation burdens obtained from a simple linear regression model fitted on the corresponding average copy number log-ratios.

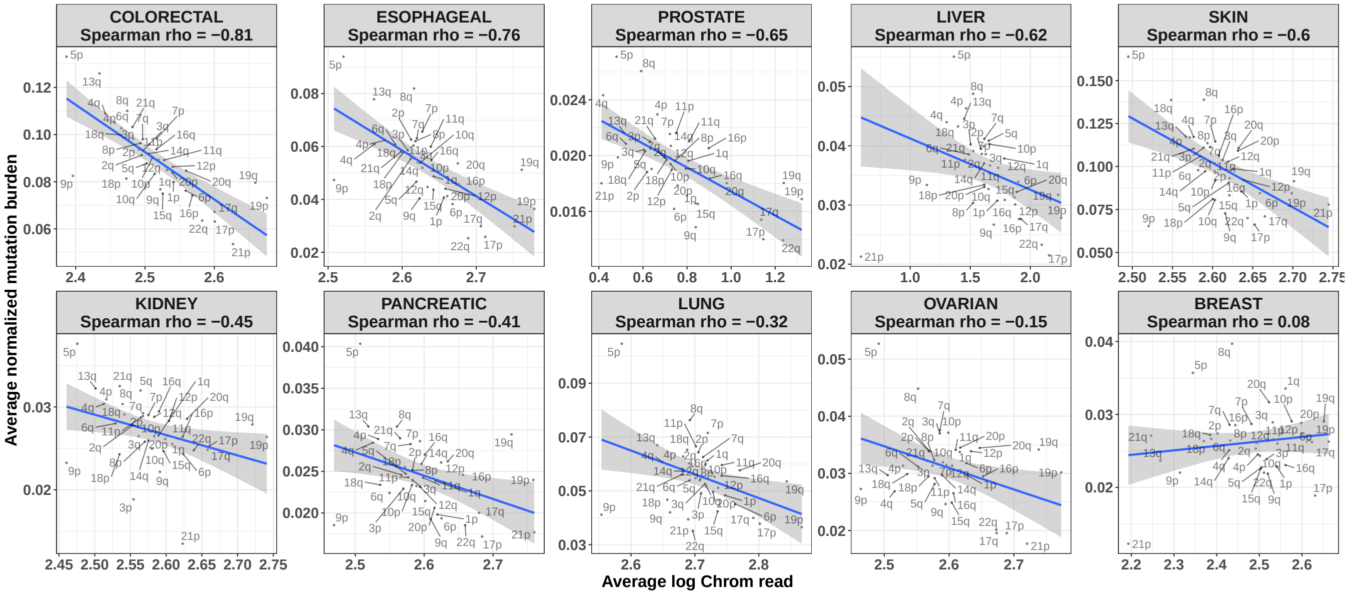

Supplementary Figure 12: Visualizing association between mutation burden and chromatin accessibility. The average normalized whole genome mutation burden in each chromosome arm is plotted against the corresponding average log chromatin accessibility reads for different cancer sites. The error band in each plot corresponds to 95% prediction intervals for average normalized mutation burdens obtained from a simple linear regression model fitted on the corresponding average log chromatin accessibility reads.

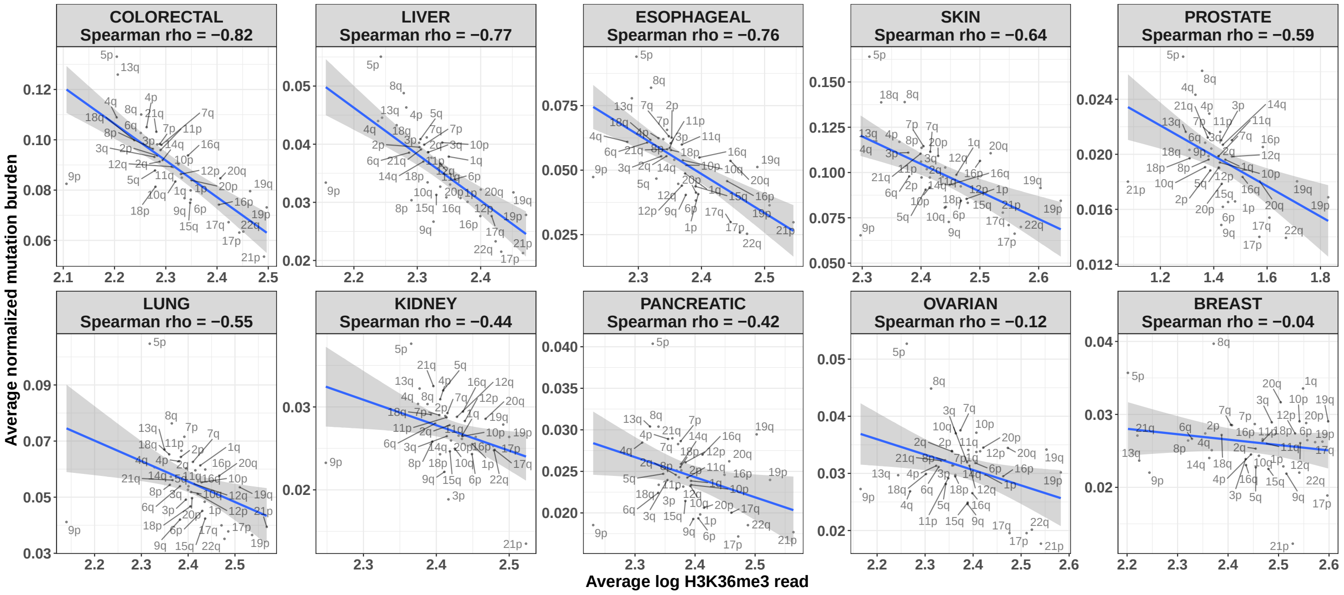

Supplementary Figure 13: Visualizing association between mutation burden and H3K36me3. The average normalized whole genome mutation burden in each chromosome arm is plotted (along the vertical axis) against the corresponding average log H3K36me3 reads (along the horizontal axis) for different cancer sites. The error band in each plot corresponds to 95% prediction intervals for average normalized mutation burdens obtained from a simple linear regression model fitted on the corresponding average log H3K36me3 reads.

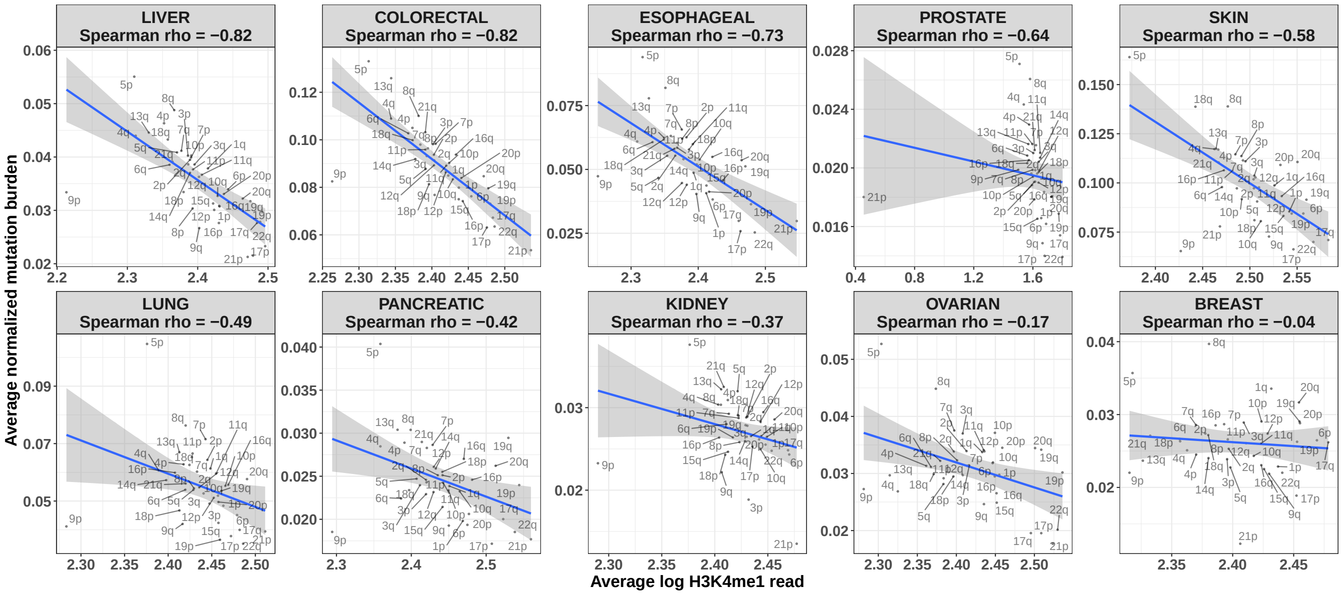

Supplementary Figure 14: Visualizing association between mutation burden and H3K4me1. The average normalized whole genome mutation burden in each chromosome arm is plotted (along the vertical axis) against the corresponding average log H3K4me1 reads (along the horizontal axis) for different cancer sites. The error band in each plot corresponds to 95% prediction intervals for average normalized mutation burdens obtained from a simple linear regression model fitted on the corresponding average log H3K4me1 reads.

Supplementary References

[1] Chakraborty, S., Martin, A., Guan, Z., Begg, C. B. & Shen, R. *hidgenclassifier: Functions for Bayesian hierarchical hidden genome classifier* (2020).

[2] Friedman, J., Hastie, T. & Tibshirani, R. glmnet: Lasso and elastic-net regularized generalized linear models. *R package version* (2009).

[3] Wright, M. N. & Ziegler, A. {ranger}: A Fast Implementation of Random Forests for High Dimensional Data in {C++} and {R}. *Journal of Statistical Software* **77**, 1–17 (2017).

[4] Steinwart, I. & Thomann, P. {liquidSVM}: A Fast and Versatile {SVM} package. *ArXiv e-prints 1702.06899* (2017). URL http://www.isa.uni-stuttgart.de/software.

[5] Allaire, J. J. & Chollet, F. *keras: {R} Interface to {'Keras'}* (2020). URL https://cran.r-project.org/package=keras.

[6] Allaire, J. J. & Tang, Y. *tensorflow: R Interface to {'TensorFlow'}* (2020). URL https://cran.r-project.org/package=tensorflow.

[7] Kuhn, M. *caret: Classification and Regression Training* (2020). URL https://cran.r-project.org/package=caret.

[8] Bischl, B. *et al.* {mlr}: Machine Learning in R. *Journal of Machine Learning Research* **17**, 1–5 (2016). URL https://jmlr.org/papers/v17/15-066.html.

[9] Bischl, B. *et al.* mlrMBO: A Modular Framework for Model-Based Optimization of Expensive Black-Box Functions. *arXiv preprint arXiv:1703.03373* (2017).

[10] Kingma, D. P. & Ba, J. L. Adam: A method for stochastic optimization. In *3rd International Conference on Learning Representations, ICLR 2015 - Conference Track Proceedings* (2015). 1412.6980.

[11] Jiao, W. *et al.* A deep learning system accurately classifies primary and metastatic cancers using passenger mutation patterns. *Nature Communications* **11**, 1–12 (2020).
