# Supplementary material for: Mining mutation contexts across the cancer genome to map tumor site of origin
Source: Nat Commun. 2021 May 24;12:3051. doi: 10.1038/s41467-021-23094-z (PMC8144407; doi:10.1038/s41467-021-23094-z)
Supplement: Supplementary file 3 — Description of Additional Supplementary Files [file 41467_2021_23094_MOESM3_ESM.pdf]

Description of Additional Supplementary Files

File Name: Supplementary Data 1
Description: Excel tables providing cancer site, histology and tumor sample sizes in PCAWG, TCGA and MSK-IMPACT datasets, and data sources for the epigenomic features used.
