# Supplementary material for: Mining mutation contexts across the cancer genome to map tumor site of origin
Source: Nat Commun. 2021 May 24;12:3051. doi: 10.1038/s41467-021-23094-z (PMC8144407; doi:10.1038/s41467-021-23094-z)
Supplement: Supplementary file 5 — Reporting Summary [file 41467_2021_23094_MOESM5_ESM.pdf]

nature research

Corresponding author(s): Ronglai Shen, Colin B. Begg

Last updated by author(s): Mar 7, 2021

Reporting Summary

Nature Research wishes to improve the reproducibility of the work that we publish. This form provides structure for consistency and transparency in reporting. For further information on Nature Research policies, see our Editorial Policies and the Editorial Policy Checklist.

Statistics

For all statistical analyses, confirm that the following items are present in the figure legend, table legend, main text, or Methods section.

n/a	Confirmed	
☐	☒	The exact sample size (*n*) for each experimental group/condition, given as a discrete number and unit of measurement
☐	☒	A statement on whether measurements were taken from distinct samples or whether the same sample was measured repeatedly
☒	☐	The statistical test(s) used AND whether they are one- or two-sided<br>*Only common tests should be described solely by name; describe more complex techniques in the Methods section.*
☐	☒	A description of all covariates tested
☐	☒	A description of any assumptions or corrections, such as tests of normality and adjustment for multiple comparisons
☐	☒	A full description of the statistical parameters including central tendency (e.g. means) or other basic estimates (e.g. regression coefficient) AND variation (e.g. standard deviation) or associated estimates of uncertainty (e.g. confidence intervals)
☒	☐	For null hypothesis testing, the test statistic (e.g. *F*, *t*, *r*) with confidence intervals, effect sizes, degrees of freedom and *P* value noted<br>*Give P values as exact values whenever suitable.*
☐	☒	For Bayesian analysis, information on the choice of priors and Markov chain Monte Carlo settings
☒	☐	For hierarchical and complex designs, identification of the appropriate level for tests and full reporting of outcomes
☐	☒	Estimates of effect sizes (e.g. Cohen's *d*, Pearson's *r*), indicating how they were calculated

Our web collection on statistics for biologists contains articles on many of the points above.

Software and code

Policy information about availability of computer code

Data collection	All data sets used in the analysis are publicly available, and were downloaded from their respective webpages upon data access approval. No software was used for data collection.
Data analysis	All statistical analyses were performed in statistical software R v4.0.2. Custom codes developed in the study are available through GitHub at: https://github.com/c7rishi/hidgenclassifier

For manuscripts utilizing custom algorithms or software that are central to the research but not yet described in published literature, software must be made available to editors and reviewers. We strongly encourage code deposition in a community repository (e.g. GitHub). See the Nature Research guidelines for submitting code & software for further information.

Data

Policy information about availability of data

All manuscripts must include a data availability statement. This statement should provide the following information, where applicable:

- Accession codes, unique identifiers, or web links for publicly available datasets
- A list of figures that have associated raw data
- A description of any restrictions on data availability

The TCGA whole exome somatic mutation data used in this study are openly available in the GDC database [https://gdc.cancer.gov/about-data/publications/mc3-2017]. The MSK-IMPACT somatic mutation data used in this study are available in the cBioPortal database [https://www.cbioportal.org/study/summary?id=msk_impact_2017]. The controlled access PCAWG whole genome sequencing datasets are deposited at the ICGC database [https://dcc.icgc.org/]. The data is available under restricted access, access can be obtained by contacting daco@icgc.org. The exact processed subsets of the TCGA and MSK-IMPACT datasets used in our analysis are included as R data objects within the custom R package hidgenclassifier25 developed in this study and released publicly through GitHub [https://github.com/c7rishi/hidgenclassifier]. In the same GitHub repository an interactive html version of Figure 1 is also stored. Individual sources for the publicly available

epigenomic data sets used for construction of meta-features are listed in Sheet 4 of the excel file provided as Supplementary Data 1. The remaining data are available within the Article or Supplementary Information are available from the authors upon request.

Field-specific reporting

Please select the one below that is the best fit for your research. If you are not sure, read the appropriate sections before making your selection.

- [x] Life sciences
- [ ] Behavioural & social sciences
- [ ] Ecological, evolutionary & environmental sciences

For a reference copy of the document with all sections, see nature.com/documents/nr-reporting-summary-flat.pdf

Life sciences study design

All studies must disclose on these points even when the disclosure is negative.

Sample size	All sample points present in the three publicly available databases, viz., PCAWG, TCGA and MSK-IMPACT, were considered in the analysis. These three datasets provide mutational information of cancer tumors at three sequencing coverage levels -- whole genome, whole exome and targeted cancer gene panel respectively. We elected to focus on 10 common cancer sites on which data were available in the three databases with at least moderate sample sizes (n >= 30). Our study is primarily methodological, and tumors sequenced in three databases are sufficient to demonstrate and exemplify methodologies developed in our study.
Data exclusions	No data points were excluded.
Replication	Our cross-validation based predictive analyses were replicated 10 times, and all replicates were considered. The average predictive accuracies obtained from all 10 replications were reported.
Randomization	This is not relevant to our study because we used publicly available databases curated from large scale genome sequencing studies to demonstrate and exemplify our novel methodologies. Randomizations are ensured in the original studies where these databases are curated.
Blinding	This is not relevant to our study because we used publicly available databases curated from large scale genome sequencing studies to demonstrate and exemplify our novel methodologies. Blindings are ensured in the original studies where these data are curated.

Reporting for specific materials, systems and methods

We require information from authors about some types of materials, experimental systems and methods used in many studies. Here, indicate whether each material, system or method listed is relevant to your study. If you are not sure if a list item applies to your research, read the appropriate section before selecting a response.

Materials & experimental systems

n/a	Involved in the study	
x		Antibodies
x		Eukaryotic cell lines
x		Palaeontology and archaeology
x		Animals and other organisms
x		Human research participants
x		Clinical data
x		Dual use research of concern

Methods

n/a	Involved in the study	
x		ChIP-seq
x		Flow cytometry
x		MRI-based neuroimaging
